# Loss of Function of von Hippel-Lindau Trigger Lipocalin 2-Dependent Inflammatory Responses in Cultured and Primary Renal Tubular Cells

**DOI:** 10.1155/2021/5571638

**Published:** 2021-06-22

**Authors:** Chan-Yen Kuo, Valeria Chiu, Po-Chun Hsieh, Tien Hsu, Ting-Yun Lin

**Affiliations:** ^1^Department of Research, Taipei Tzu Chi Hospital, Buddhist Tzu Chi Medical Foundation, New Taipei City, Taiwan; ^2^Department of Physical Medicine and Rehabilitation, Taipei Tzu Chi Hospital, Buddhist Tzu Chi Medical Foundation, New Taipei City, Taiwan; ^3^Department of Chinese Medicine, Taipei Tzu Chi Hospital, Buddhist Tzu Chi Medical Foundation, New Taipei City, Taiwan; ^4^Department of Biomedical Sciences and Engineering, National Central University, Jhongli, Taiwan; ^5^Institute of Systems Biology and Bioinformatics, National Central University, Jhongli, Taiwan; ^6^Division of Nephrology, Taipei Tzu Chi Hospital, Buddhist Tzu Chi Medical Foundation, and School of Medicine, Tzu Chi University, Hualien, Taiwan

## Abstract

Previous studies have shown that mutations in the tumor suppressor gene von Hippel-Lindau (*VHL*) can result in the overproduction of reactive oxygen species (ROS) and chronic inflammation and are a significant predisposing factor for the development of clear-cell renal cell carcinoma (ccRCC). To study VHL's role in ccRCC formation, we previously developed a novel conditional knockout mouse model that mimicked the features of kidney inflammation and fibrosis that lead to cyst formation and hyperplasia. However, due to VHL's complex cellular functions, the mechanism of this phenomenon remains unclear. Here, we used the HK-2 cells and mouse primary renal tubule cells (mRTCs) carrying *VHL* mutations as models to study the effects and underlying molecular mechanisms of ROS accumulation. We also studied the role of lipocalin 2 (LCN2) in regulating macrophage recruitment by HK-2 cells. We measured the level of ROS in HK-2 cells in the presence or absence of LCN2 knockdown and found that the *VHL* mutation caused ROS overproduction, but an LCN2 knockdown could attenuate the process. VHL was also found to mediate the *in vitro* and *in vivo* expression and secretion of LCN2. Thus, VHL likely affects ROS production in an LCN2-dependent manner. Our findings also suggest that LCN2 sensitizes the inflammatory response of HK-2 cells and the chemotactic abilities of macrophage RAW264.7 cells. By demonstrating that the loss of function of von Hippel-Lindau triggers lipocalin 2-dependent inflammatory responses in cultured and primary renal tubular cells, our results offer novel insights into a potential therapeutic approach for interfering with the development of ccRCC.

## 1. Introduction

Mutations in von Hippel-Lindau (VHL) play a critical role in developing clear-cell renal cell carcinoma (ccRCC) [1, 2]. Our previous study showed that, in the kidney lesions in *Vhl* knockout mice, there was a marked increase in inflammation and fibrosis with substantial collagen fiber deposition accumulated within clusters of distorted tubules [3]. We have previously demonstrated that the inflammation and fibrosis in *Vhl* knockout mouse lesions can be alleviated after treatment with an inhibitor of serine/threonine protein kinase/endoribonuclease (IRE1*α*) [4]. Inflammation may be caused by changes in proteostasis due to the overproduction of inflammatory cytokines [5, 6]. Notably, it has been hypothesized that inflammation plays a critical role in carcinogenesis [7], and *VHL* mutation-associated ccRCC likely results from chronic inflammation [3, 8].

Also, VHL is known to regulate many other genes involved in the inflammatory response. For example, VHL regulates the expression of *MKI67*, a *HIF1Α* target gene [9], and the marker of ccRCC [10], *CA9*, whose expression in kidney lesions may also be involved in the pathogenesis of ccRCC.

We further elucidated the inflammatory response in the *Vhl* knockout kidney by performing a microarray to profile the genes in the whole-kidney extract (Gene Expression Omnibus accession number GSE116326). We used the whole-kidney extract instead of the isolated tubule cell extract to examine the changes in the reactive microenvironment in the *Vhl* mutant cells. The microarray data showed that one of the candidate genes, *LCN2*, which encoded lipocalin 2, was highly overexpressed in the *Vhl* mutant kidney.

Lipocalin 2, also known as neutrophil gelatinase-associated lipocalin (NGAL), oncogene 24p3, uterocalin, or siderocalin, is a 24 kDa secreted glycoprotein initially purified from mouse kidney cells infected with simian virus 40 (SV-40) [11]. LCN2 mediates several cellular processes, including apoptosis, proliferation, epithelial-to-mesenchymal differentiation, and matrix metalloproteinase 9 stabilization [12]. High levels of *LCN2* expression have been observed in renal, breast, ovary, colon, and brain cancer cells [13–15]. In addition, LCN2 is a member of the lipocalin superfamily of proteins that transport hydrophobic molecules, such as retinoids, fatty acids, and organic iron chelators [16]. There is also growing evidence that LCN2 exhibits a cellular protective effect by ameliorating oxidative stress-mediated toxicity under harmful conditions, such as ROS accumulation [17–19].

This study sought to investigate the potential role of LCN2 in mediating inflammatory response in *VHL*-mutated HK-2 cells. Furthermore, we tested the hypothesis that LCN2 was related to *VHL* mutation-sensitized macrophage migration and mediated inflammation via LCN2-ROS-dependent pathways.

## 2. Materials and Methods

### 2.1. Reagents

Liproxstatin-1 (Sigma, MO, USA) and 4′,6-diamidino-2-phenylindole (DAPI, Thermo Fisher Scientific, MA, USA) were purchased.

### 2.2. Cell Culture

HK-2 (human renal proximal tubular epithelial) and HEK293 (human cell line of kidney origin) cells (Bioresource Collection and Research Center, Taiwan) were cultured in T75 flasks (Corning, NY, USA) in DMEM/Ham's F12 (Gibco, NY, USA) supplemented with 10% fetal bovine serum, 25 mM D-glucose, 2 mM L-glutamine, 1 mM sodium pyruvate, and penicillin-streptomycin (50 U/mL; Sigma, MO, USA) at 37°C in a 5% CO_2_/95% air incubator. The fresh culture medium was replaced every other day. Once the cells reached 60–70% confluence, they were trypsinized for the following experiments.

The murine monocyte/macrophage cell line RAW264.7 (Bioresource Collection and Research Center, Taiwan) was cultured in T75 flasks in Dulbecco's Modified Eagle's Medium (DMEM, Gibco, NY, USA) supplemented with 10% fetal bovine serum, 4 mM L-glutamine, 4500 mg/L glucose, 1 mM sodium pyruvate, 1500 mg/L sodium bicarbonate, and penicillin-streptomycin (50 U/mL; Sigma, MO, USA) at 37°C in a 5% CO_2_ incubator. The fresh culture medium was replaced every other day. Once the cells reached 50–60% confluence, they were trypsinized for the following experiments.

### 2.3. Culture of Primary Renal Tubular Epithelial Cells

Mouse primary proximal tubule cells (mRTCs) were isolated from wild-type *Hoxb7-Cre-GFP/+* (W) or mutant *Hoxb7-Cre-GFP/+*; *Vhl^fl/fl^* (M) mice using a previously described method [20–22], with some modifications. Briefly, mice were sacrificed by cervical dislocation. The kidneys were immediately removed and placed in 15 mL conical tubes with ice-cold Hank's balanced salt solution (Biological Industries, CT, USA). The renal capsule, cortex, and excess fat were removed, and the remaining cortical tissue was added into the dunce using two razors. Next, the plunger was pushed down to the bottom of the glass five times to break up the tissue. Then, the tissues were transferred into a 50 mL conical tube on ice. The tubular tissues were centrifuged in a swinging bucket rotor at 500 rpm and 4°C for 2 minutes. Then, the supernatant was aspirated and discarded while leaving the pelleted tissue intact.

Afterward, the tube was filled with warm digestion medium containing Collagenase I (Worthington, NJ, USA) at 140 units/mL and 15 mg Soybean Trypsin Inhibitor (Sigma, MO, USA) in 20 mL HBSS and incubated on an orbital shaker at 70 rpm for 15 minutes at 37°C. The tubule suspension was mixed with a 10 mL pipet and returned to the incubator every 5 minutes. After digestion, the tubule suspension was incubated with 20 mL of cold horse serum to inactivate the enzymes and enrich the tubules; the tube was inverted until the suspension became uniform. Then, the tubules were allowed to settle for one minute. The supernatant containing the proximal tubules was transferred to a 50 mL conical tube and centrifuged for 2 minutes at 500 rpm in a swinging bucket rotor. The tubules were washed with 10 mL of HBSS and centrifuged at 500 rpm for 2 minutes. A volume of 1–2 mL of culture medium was added to mix gently with the pellet using a sterile pipet. The cortical tubule suspension was gently layered onto a preformed 40% Percoll/60% culture medium gradient. Then, the tubules were centrifuged at 400 × g for 10 min at 4°C. The proximal renal tubules in the largest band were transferred to a 50 mL conical tube containing 20 mL of culture medium and centrifuged at 500 rpm for 2 minutes.

Finally, the tubule pellet was resuspend in 20 mL of MRPTC Culture Medium, which consisted of DMEM/F-12 culture media (Gibco, NY, USA) with insulin/transferrin/selenium (5 *μ*g/mL, 5 *μ*g/mL, and 5 ng/mL, respectively, Sigma), 0.05 *μ*M hydrocortisone (Sigma), 50 *μ*M L-ascorbic acid-2-phosphate (Wako, Tokyo, Japan), and 1% antibiotic/antimycotic solution (10,000 units/mL penicillin, 0.1 mg/mL streptomycin, and 0.25 *μ*g/mL amphotericin B, Biological Industries, CT, USA).

Dilutional studies were performed to determine the volume of the resuspended tubules needed for different plates to ensure optimal growth. The tubules were plated on 12-well (1–1.5 mL/well) or 6-well (3 mL/well) Nunclon-treated tissue culture plates (Nalgene/Nunc International, Rochester, NY) and incubated at 37°C with 5% CO_2_. The media was replaced with MRPTC culture media every day. Confluence can be achieved in 5 to 12 days.

### 2.4. Transfection

HK-2 cells were transfected with a vector with a scrambled sequence or a vector expressing shVHL1 (V1), shVHL3 (V3), or shLCN2 (L1) (kindly provided by Dr. T. Hsu, Department of Biomedical Sciences and Engineering, National Central University, Jhongli, Taiwan) using the BTX™ Gemini X2 Electroporation System (BTX, MA, USA), according to the manufacturer's protocol. Briefly, 5 × 10^5^ cells were transfected with 4 *μ*g of a plasmid with a 100 V pulse for 10 msec, plated in a 6-well plate, and cultured for 48 hours before being analyzed for *VHL* expression by Western blot to determine the transfection efficiency.

### 2.5. Measurement of Intracellular ROS Generation

The analysis of the measurement of intracellular ROS generation was according to our previous studies with some modifications [23]. The cells were washed with phosphate-buffered saline (PBS) and incubated with 10 *μ*M dihydroethidium (DHE; Santa Cruz, Dallas, TX, USA) in Hank's balanced salt solution at 37°C for 30 minutes in the dark. During incubation with ROS, DHE is oxidized and becomes fluorescent. After incubation, the cells were trypsinized and washed with ice-cold PBS three times. The level of ROS was quantified by flow cytometry (BD Biosciences, San Jose, CA, USA).

### 2.6. Western Blot Analyses

The antibodies for Western blot analyses included the mouse monoclonal antibodies against VHL (BD Biosciences, Franklin Lakes, NJ, USA); NF-*κ*B's p65 subunit (GeneTex, Irvine, CA, USA); JNK (Cell Signaling, MA, USA); p-JNK, *β*-actin, and fibrillarin (Santa Cruz, Dallas, TX, USA); and the rabbit polyclonal antibodies against LCN2 and GAPDH (ABclonal, MA, USA). The *β*-actin was diluted 1 : 500 in the final working solution; all other antibodies were diluted 1 : 1000.

### 2.7. Nuclear Fraction Extraction

The chemotaxis analysis was according to our previous studies with some modifications [23]. Nuclear fractions were extracted from the HK-2 or mRTC cells. The cells were collected and resuspended in a hypotonic buffer (10 mM HEPES, pH 7.9; 10 mM KCl; 1.5 mM MgCl_2_; 0.2 mM PMSF; 20 *μ*g/mL aprotinin; 0.5 mM DTT; and 0.5% NP-40) on ice for 15 minutes. After centrifuging at 6000 × g for 15 minutes at 4°C, the pellet was collected and washed with a basal buffer (the hypotonic buffer without the 0.5% NP-40). After centrifuging again at 6000 × g for 15 minutes at 4°C, the pellet was collected and resuspended in a hypertonic buffer (20 mM HEPES, pH 7.9; 400 mM KCl; 1.5 mM MgCl_2_; 0.2 mM PMSF; 20 *μ*g/mL aprotinin; 0.5 mM DTT; 0.2 mM EDTA; 10% glycerol) at room temperature for 30 minutes. After centrifuging at 10,000 × g for 30 minutes at 4°C, the supernatant containing the nuclear fraction was collected.

### 2.8. Chemotaxis Assay

The chemotaxis analysis was according to our previous studies with some modifications [23]. A 24-well Transwell plate (8 *μ*m pore size, Corning, NY, USA) was used to measure the chemotactic ability of the RAW274.7 cells. HK-2 cells were added into the lower chamber and transfected with a vector carrying a scrambled or the shVHL [24] sequence by electroporation. After incubation for 48 hours, the cells were treated with the indicated inhibitors for 24 hours in a serum-free medium supplemented with 5% bovine serum albumin (BIONOVAS, Toronto, Canada). Then, 1 × 10^5^ RAW264.7 cells were added to the upper chamber with an uncoated membrane in a serum-free medium supplemented with 5% bovine serum albumin (BIONOVAS, Toronto, Canada). In separate experiments, RAW264.7 cells were exposed to recombinant human lipocalin 2/NGAL protein (rhLCN2) (Novus Biologicals, CO, USA) added to the serum-free medium and their migration measured. After 24 hours, the migratory cells on the underside of the membrane of the upper chamber were stained with 0.1% crystal violet for 5 minutes, washed with H_2_O, and scanned using an EPSON V750 PRO scanner. The cells were destained for crystal violet with methanol for 15 minutes and measured at OD570 with the Synergy HT (BioTek, VT, USA).

### 2.9. Statistical Analysis

The data were expressed as means ± SEM. The groups were compared using a one-way or two-way ANOVA followed by the Bonferroni post hoc analysis; *p* < 0.05 was considered statistically significant.

## 3. Results

### 3.1. *VHL* Mutation-Induced ROS Production in an LCN2-Dependent Manner

**Figure 1:**
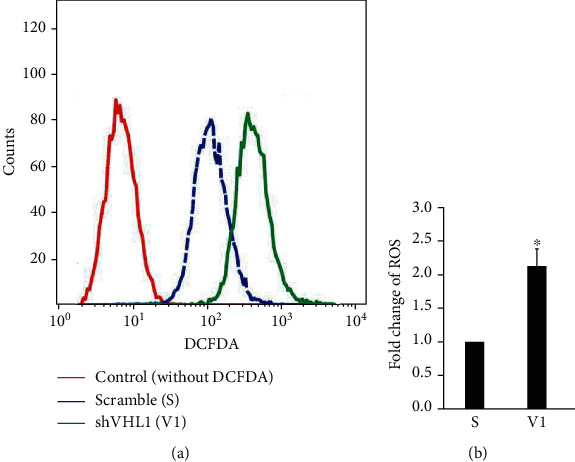
Effect of VHL on ROS production in HK-2 cells. HK-2 cells were transfected with a vector expressing a scrambled shRNA (SC) or shRNA specific for VHL (shVHL1) (V1) (4) by electroporation with the Gemini X2 System and incubated for 48 hours. (a) After transfection, the intracellular ROS levels were determined using DCFDA. (b) The fluorescence intensity was detected using FACSCalibur analysis. All data were presented as the mean ± SD. ^∗^*p* < 0.05 as compared to the scrambled vector.

We investigated the effect of *VHL* deficiency on ROS production by measuring the level of ROS in *VHL* knockdown HK-2 cells using a previously described protocol [25–27]. We found that *VHL* knockdown caused ROS overproduction in HK-2 cells (Figure 1).

**Figure 2:**
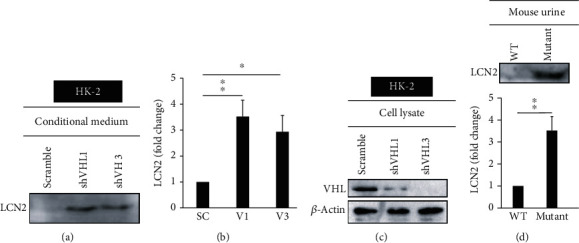
*VHL* knockdown induced LCN2 secretion in HK-2 cells. HK-2 cells were transfected with a vector expressing a scrambled shRNA (SC) or an shRNA specific for VHL: shVHL1 (V1) or shVHL3 (V3), as described in a previous study (4) by electroporation (Gemini X2 System) and incubated for 48 hours. (a) The Western blot analysis of LCN2 using an anti-LCN2 antibody at 50 *μ*g of protein/lane in the conditioned medium under the indicated conditions in the HK-2 cells. (b) The level of LCN2 was quantified using ImageJ. (c) The cell lysates were subject to Western blot analysis with the anti-LCN2 antibody. (d) An equal volume of 10 *μ*L of urine samples from the wild-type and *Vhl* conditional knockout mice was analyzed by Western blot analysis using the anti-LCN2 antibody. The lower panel shows the level of LCN2 was quantified by ImageJ. All data are presented as the mean ± SD. ^∗^*p* < 0.05 and ^∗∗^*p* < 0.01.

**Figure 3:**
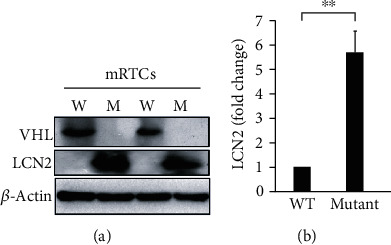
LCN2 was increased in *Vhl* mutant mRTCs. (a) Primary mouse renal tubule cells (mRTCs) were isolated from the wild-type *Hoxb7-Cre-GFP/+* (W) or the mutant *Hoxb7-Cre-GFP/+*; *Vhl^fl/fl^* (M) mice. The cell lysate was subject to Western blot analysis with the indicated antibodies. *β*-Actin was used as a loading control. The knockout of *Vhl* was confirmed by assaying the level of VHL (top). (b) The level of LCN2 was quantified using ImageJ. All data are presented as the mean ± SD. ^∗∗^*p* < 0.01.

LCN2 is one of the candidate genes predominantly related to immune response; however, its role in precancerous kidney cells is still unclear. We hypothesized that *VHL* deficiency caused inflammation and immune cell infiltration by overexpressing *LCN2* in HK-2 cells and mouse primary renal tubule cells (mRTCs). *VHL* knockdown was found to induce LCN2 secretion in HK-2 cells (Figures 2(a) and 2(b)). Meanwhile, the level of LCN2 was elevated in *Vhl* mutant mRTCs (Figure 3). Interestingly, the enhanced level of LCN2 was detected in the urine of *Vhl* conditional knockout mice (Figure 2(d)). The presence of LCN2 in urine indicates acute kidney injury (AKI) [28]. These findings demonstrated that VHL deficiency induced expression and secretion of LCN2 *in vitro* and *in vivo*.

**Figure 4:**
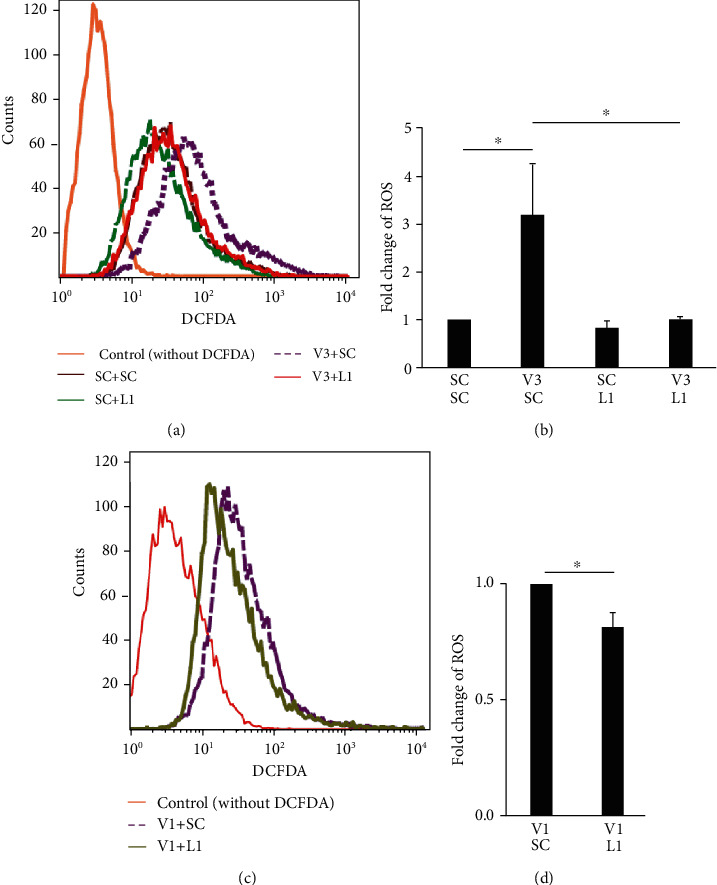
*LCN2* knockdown attenuated the increase in ROS production in *VHL* knockdown HK-2 cells. (a) The change in the level of cellular ROS in HK-2 cells transfected with a vector expressing one scrambled shRNA (SC), two scrambled shRNAs (SC+SC), the scrambled shRNA (SC) and shRNA specific for LCN2 (L1) (SC+L1), the shRNA specific for VHL (V3) and scrambled shRNA (SC) (V3+SC), or the shRNA specific for VHL (V3) and shRNA specific for LCN2 (L1) (V3+L1). (b) ROS generation was expressed as mean fluorescence intensity. (c) The change in the level of cellular ROS in HK-2 cells transfected with a vector expressing the shRNA specific for VHL (V1) and scrambled shRNA (SC) (V1+SC) or shRNA specific for VHL (V1) and shRNA specific for LCN2 (L1) (V1+L1). (d) ROS generation was expressed as mean fluorescence intensity. All data are presented as the mean ± SD. ^∗∗^*p* < 0.01.

We further studied the role of LCN2 on ROS production by examining the effect of *LCN2* knockdown on ROS levels. The increase in ROS levels was attenuated in *LCN2* knockdown in *VHL*-deficient (knockdown) HK-2 cells (Figure 4).

### 3.2. *Vhl* Mutant Caused Inflammatory Response and Sensitized RAW264.7 Cell Chemotaxis in a LCN2-Dependent Manner

**Figure 5:**
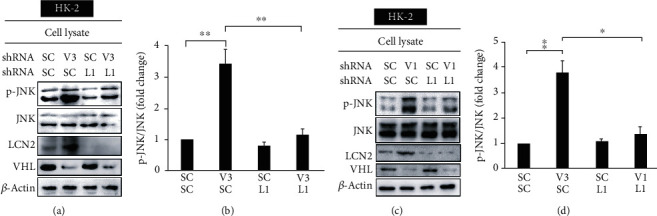
VHL loss of function exhibited an inflammatory response in an LCN2-dependent manner. (a) HK-2 cells were transfected with a vector expressing a scrambled shRNA (SC), an shRNA specific for VHL: shVHL1 (V1), or shLCN2 (L1). Cell lysates were analyzed with Western blot using the indicated antibodies. *β*-Actin was used as a loading control. The status of LCN2 and VHL was confirmed by assaying the levels of LCN2 and VHL proteins, respectively. The *VHL* knockdown increased the level of p-JNK, but the increase was attenuated in *VHL* (V3) and *LCN2* (L1) double knockdown cells. (b) The level of p-JNK was quantified using ImageJ. (c) HK-2 cells were transfected with a vector expressing the scrambled shRNA (SC), shRNA specific for VHL: shVHL3 (V3), or shLCN2 (L1). The cell lysates were analyzed by Western blot using the indicated antibodies. *β*-Actin was used as a loading control. The knockdown of *LCN2* and *VHL* was confirmed by assaying the levels of LCN2 and VHL proteins, respectively. *VHL* knockdown increased the level of p-JNK, but the increase was attenuated in *VHL* (V1) and *LCN2* (L1) double knockdown cells. (d) The level of p-JNK was quantified using ImageJ. All data are presented as the mean ± SD. ^∗^*p* < 0.05 and ^∗∗^*p* < 0.01.

**Figure 6:**
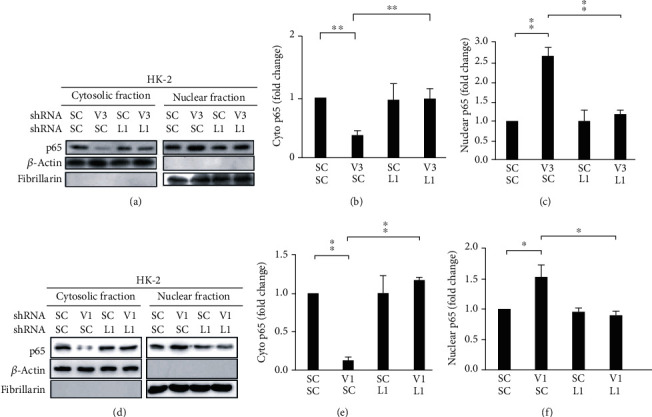
VHL loss of function caused the nuclear translocation of p65 in an LCN2-dependent manner. (a) The HK-2 cells were cultured and treated, as described in Figure 4. Their cell lysates were collected and separated into cytosolic and nuclear fractions. The subcellular localization of NF-*κ*B was detected by Western blot using an antibody against NF-*κ*B's subunit p65. *β*-Actin was used as a cytosolic marker and fibrillarin as a nuclear marker. (b) NF-*κ*B's cytosolic localization was decreased in *VHL* knockdown (V3) cells; such decrease was attenuated in the *VHL* (V3) and *LCN2* (L1) double knockdown cells. The level of cytosolic p65 (cyto p65) was quantified using ImageJ. (c) NF-*κ*B's nuclear localization was increased in *VHL* knockdown (V3) cells; however, such an increase was attenuated in *VHL* (V3) and *LCN2* (L1) double knockdown cells. The expression of nuclear p65 (nuclear p65) was quantified using ImageJ. (d) The HK-2 cells were cultured and treated, as described in Figure 4. The cell lysates were collected and separated into cytosolic and nuclear fractions. The subcellular localization of NF-*κ*B was detected with Western blot using an antibody against the NF-*κ*B subunit p65. *β*-Actin was used as a cytosolic marker and fibrillarin as a nuclear marker. (e) NF-*κ*B's cytosolic localization was decreased in VHL knockdown (V1) cells; however, the decrease was attenuated in *VHL* (V1) and *LCN2* (L1) double knockdown cells. The level of cytosolic p65 (cyto p65) was quantified using ImageJ. (f) NF-*κ*B's nuclear localization was increased in VHL knockdown (V1) cells; the increase was attenuated in *VHL* (V1) and *LCN2* (L1) double knockdown cells. The level of nuclear p65 (nuclear p65) was quantified using ImageJ. All data are presented as the mean ± SD. ^∗^*p* < 0.05 and ^∗∗^*p* < 0.01.

We dissected the role of LCN2 in inflammation by investigating the effect of *LCN2* knockdown on the phosphorylation of JNK and the nuclear translocation of NF-*κ*B's p65 subunit in HK-2 cells and chemotaxis of RAW264.7 cells. *LCN2* knockdown was found to significantly attenuate the increase in p-JNK expression activated by Figure 5. *LCN2* knockdown also diminished the nuclear translocation of p65 in the *VHL* knockdown HK-2 cells, as demonstrated by Western blotting (Figure 6).

**Figure 7:**
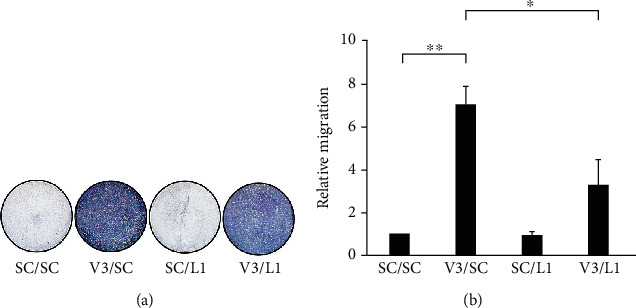
*LCN2* knockdown attenuated the VHL inactivation-induced chemotaxis of macrophages. The HK-2 cells or without the knockdown of *VHL* (V3) or without (SC) and with knockdown of *LCN2* (L1) or without (SC) were assayed for their ability to recruit monocyte/macrophage RAW264.7 cells. (a) HK-2 cells transfected with vectors containing a scrambled shRNA sequence (SC), shVHL3 (V3), or shLCN2 (L1) were assayed for their respective capacity to induce macrophage chemotaxis for 48 hours. The macroscopic observation of the Transwell chambers was performed. (b) Migration in the Transwell system was allowed for 24 hours. The relative number of migrated cells stained by crystal violet was measured at OD_570_. All data were presented as the mean ± SD. ^∗^*p* < 0.05 and ^∗∗^*p* < 0.01.

**Figure 8:**
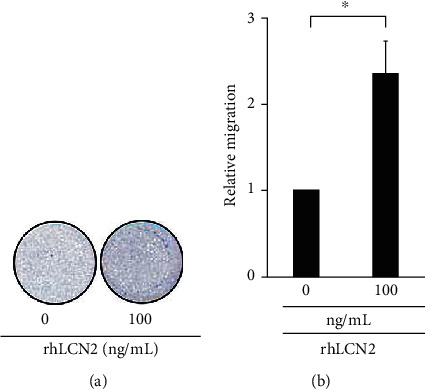
LCN2 significantly induced macrophage cell migration. (a) The treatment of human recombinant LCN2 (rhLCN2) at 100 ng/mL increased RAW264.7 cell migration compared to no (0 ng/mL rhLCN2) treatment. (b) Migration in the Transwell system was allowed for 24 hours. The relative number of migrated cells stained by crystal violet was measured at OD_570_ is shown. All data are presented as the mean ± SD. ^∗^*p* < 0.05.

On the other hand, *Vhl* mutant was found to induce the chemotaxis of the RAW264.7 cells (Figure 7, V3/SC compared with SC/SC), and LCN2 knockdown could reduce the Transwell migration of RAW264.7 induced by VHL knockdown HK-2 cells (Figure 7, V3/L1 compared with V3/SC). This suggests that LCN2 is required for the chemotactic function of *VHL*-deficient HK-2 cells. We confirm the chemotactic effect of LCN2 by comparing the relative migration of RAW264.7 cells in the absence and presence (100 ng/mL) of recombinant human LCN2 (rhLCN2). We found that LCN2 significantly induced RAW264.7 cell migration after the rhLCN2 treatment (Figure 8). In summary, our findings suggested that LCN2 sensitizes the inflammatory response of HK-2 cells and chemotactic abilities of macrophage RAW264.7 cells.

## 4. Discussion

The mechanism underlying the inflammatory response to *VHL* inactivation in kidney tubule cells presents an intriguing pathophysiological question and a potential therapeutic target. It has been shown that *VHL* mutant cells are involved in increased protein synthesis and ROS accumulation [4], likely due to the increased mTOR signaling in the mutant cells [29, 30]. Moreover, the significantly elevated activation of immune cells likely contributes substantially to tissue damage during inflammatory diseases.

*VHL*/*Vhl* mutation causes *HIF* overexpression [31, 32]. During *HIF* overexpression, excessive protein synthesis occurs, leading to metabolic problems, ROS accumulation, and, eventually, ER stress. In addition, *HIF* overexpression resulted in mitochondrial damage, disruption of the TCA cycle, severe hypoxia stress, and Warburg effect [30, 33].

LCN2 is critical for the inflammation in retinal degeneration [34], ocular disease [35], intestinal tract [16, 36], ischemic stroke [37], psoriasis [38], cardiovascular diseases [39], alcoholic steatohepatitis [40, 41], nonalcoholic steatohepatitis (NASH) [42], muscle-skeletal disorders [43], and lung infection [44]. However, LCN2's role in the *VHL*-mutation-mediated progression of tumor formation via the regulation of oxidative homeostasis and mitochondrial metabolism has not been previously studied. The present study shows that mutant *VHL* induces ROS production in an LCN2-dependent manner (Figures 1–4). On the other hand, the results also show that mutant *VHL* sensitized RAW264.7 cell chemotaxis in an LCN2-dependent manner (Figures 5–8). The diverse functional roles of LCN2 were exemplified in the central nervous systems [45]. Therefore, emerging evidence suggests that LCN2 has protective as well as pathogenic activities [46–48]. We propose that the dual biological effects of LCN2 may be considered during the design of therapeutics against ccRCC.

## 5. Conclusion

**Figure 9:**
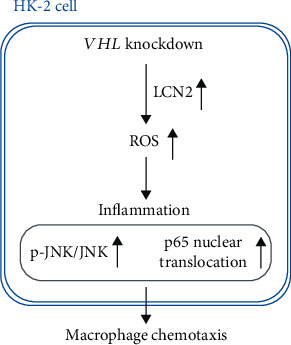
Scheme of the mechanisms. Knockdown of VHL gene in HK-2 cells induces LCN2 overexpression and ROS accumulation, leading to inflammation with increased p-JNK/JNK ratio, p65 nuclear translocation, and macrophage chemotaxis. The results reveal the regulatory effect of VHL on inflammation in HK-2 cells via the LCN2-ROS pathway.

Our results indicated that *VHL* deficiency caused overproduction of reactive oxygen species (ROS), but an LCN2 knockdown could reverse this process. *VHL* deficiency was also found to increase *in vitro* and *in vivo* LCN2 expression and secretion. Our findings reveal that the regulatory effect of *VHL* on chronic inflammation in ccRCC progression is likely mediated, at least in part, via the LCN2-ROS pathway (Figure 9). Our study offers novel insights into the therapeutic target and strategy for attenuating the development of ccRCC.

## Data Availability

The original data used to support the findings of this study are included in the article.
